# Integrating digital and narrative medicine in modern healthcare: a systematic review

**DOI:** 10.1080/10872981.2025.2475979

**Published:** 2025-05-06

**Authors:** Efthymia Efthymiou

**Affiliations:** College of Interdisciplinary Studies, Zayed University, Abu Dhabi Campus, Abu Dhabi, UAE

**Keywords:** Narrative medicine, healthcare education, medical technology, empathy, professional development, Patient-Centered care

## Abstract

The increasing integration of digital technologies in healthcare, such as electronic health records, telemedicine, and diagnostic algorithms, improved efficiency but raised concerns about the depersonalization of care. Narrative medicine has emerged as a pedagogical and clinical response to this shift, emphasizing the value of patient stories, socio-cultural contexts, and reflective practice. Understanding how digital tools support, rather than undermine, narrative competencies is critical for developing a more human-centered healthcare education. This review systematically examined empirical and theoretical studies on the integration of digital technologies and narrative medicine within healthcare education. Databases including PubMed, MEDLINE, and Google Scholar were searched using defined inclusion and exclusion criteria. Studies were screened, reviewed, and thematically analyzed to identify patterns in outcomes, pedagogical applications, and integration strategies.

The findings indicate that narrative medicine nurtures empathy, communication, and professional identity formation among healthcare trainees. Digital tools, such as virtual reality simulations, mobile health applications, and e-portfolios, reinforce these outcomes by providing immersive, interactive, and reflective learning experiences. The integration of narrative methods into digital platforms and curricular models provides a promising approach for linking clinical competence with relational care. The convergence of digital and narrative medicine provides a compelling pedagogical framework for healthcare education. This integrated approach supports technological proficiency and humanistic values, for advancements in digital health to improve rather than displace the interpersonal foundations of patient care. Further empirical research might assess long-term outcomes and guide implementation into curricula, faculty development, and institutional policy.

Background: The increasing integration of digital technologies in healthcare, such as electronic health records, telemedicine, and diagnostic algorithms, has improved efficiency but raised concerns about the depersonalization of care. Narrative medicine has emerged as a pedagogical and clinical response to this shift, emphasizing the value of patient stories, socio-cultural contexts, and reflective practice. Objective: To understand how digital tools can support, rather than undermine, narrative competencies and contribute to a more human-centered healthcare education. Methods: This review systematically examined empirical and theoretical studies on the integration of digital technologies and narrative medicine within healthcare education. Databases including PubMed, MEDLINE, and Google Scholar were searched using defined inclusion and exclusion criteria. Identified studies were screened, reviewed, and thematically analyzed to extract patterns in outcomes, pedagogical applications, and integration strategies. Results: The findings indicate that narrative medicine nurtures empathy, communication, and professional identity formation among healthcare trainees. Digital tools, including virtual reality simulations, mobile health applications, and e-portfolios, reinforce these outcomes by providing immersive, interactive, and reflective learning experiences. Integrating narrative methods into digital platforms and curricular models presents a promising approach for linking clinical competence with relational care. Conclusion: The convergence of digital and narrative medicine offers a compelling pedagogical framework for healthcare education. This integrated approach supports both technological proficiency and humanistic values, enabling advancements in digital health to enhance rather than displace the interpersonal foundations of patient care. Further empirical research is needed to assess long-term outcomes and guide implementation in curricula, faculty development, and institutional policy.

## Introduction

The rise of digital technologies in healthcare has changed clinical practices, improving efficiency, diagnostic precision, and access to care. However, this technological emphasis comes at the expense of personal interactions between healthcare providers and patients. With the widespread adoption of electronic health records (EHRs), telemedicine, and algorithmic diagnostics, concerns about the depersonalization of care are prominent. This shift stresses the need for a pedagogical and clinical balance that cultivates technical competence and relational sensitivity.

In response to the challenges of clinical detachment in an era dominated by digital tools, narrative medicine provides a vital counterbalance. This approach integrates patients’ lived experiences, cultural contexts, and emotional narratives into their medical care, encouraging therapeutic relationships that transcend technological barriers. Studies by [1,2], support the effectiveness of narrative medicine in improving communication skills and empathy, essential dimensions of comprehensive, person-centered care.

[3], highlights the value of narrative practices in tailoring care for elderly populations, while [4], demonstrates their efficacy in improving engagement among patients. Similarly [5], emphasizes the diagnostic value of patient narratives, in supportive and palliative care environments. These studies demonstrate how narrative approaches humanize care across diverse clinical settings. In this review, narrative medicine is understood to encompass reflective writing, patient storytelling, narrative-based communication training, and the cultivation of narrative competence, defined as the ability to absorb, interpret, and act upon patient stories. These components represent both pedagogical strategies and clinical tools to strengthen empathy, ethical awareness, and person-centered care in healthcare education.

Simultaneously, the digital revolution, chronicled by [6,7], continues to reshape global healthcare delivery. The accelerated deployment of telehealth platforms during the COVID-19 pandemic exemplifies the rapidly growing intersection between technology and clinical practice. In this context, the challenge is no longer whether digital tools will be adopted, but how they augment rather than erode humanistic care.

As digital technologies are integrated in the practice of medicine, the role of narrative medicine becomes essential. [8,9], argue that narrative methods improve diagnostic accuracy by contextualizing the clinical data provided by technology, increasing decision-making without compromising patient-centeredness and ensuring that technological advancements are tools for understanding, not substitutes for it. Ethical considerations also accompany the rise of big data and algorithmic decision-making. [10], alongside [11], highlight the dual-edged nature of digital health; it provides powerful capabilities but risks undermining the physician-patient relationship. Narrative medicine, functions as a safeguard, providing the interpretive and ethical frameworks needed to navigate these technological shifts responsibly.

Digital health redefines clinical care and medical education. As [12,13,14], note, the integration of patient-centered digital tools is already reconfiguring healthcare workflows. However, without a deliberate integration of narrative frameworks, these innovations may be insufficient to support the holistic care that modern patients require. While several prior studies have explored narrative medicine or the impact of digital health tools in isolation, few reviews have examined their combined pedagogical potential. This review addresses this gap by synthesizing evidence on their integration within healthcare education.

The review focuses primarily on applications in undergraduate and postgraduate medical education, including narrative-based clinical training and digital instructional innovations. It also incorporates theoretical perspectives from narrative pedagogy and digital learning to support its analysis. Moreover, it explores the coactive integration of digital and narrative medicine within healthcare education, a convergence that advances empathy, communication, professionalism, and digital fluency. Through empirical research and theoretical models, this paper outlines strategic pathways for implementation and identifies areas requiring further empirical validation to provide a blueprint for a healthcare education system that is technologically advanced and overpoweringly human-centered.

## Materials and methods

To conduct this systematic review, we employed a detailed and structured search strategy aimed at identifying relevant studies that examine the role of narrative medicine within the context of modern healthcare education, especially considering the rise of digital technologies. The objective of this systematic review was to critically evaluate the integration of narrative medicine and digital tools within healthcare education, with a particular focus on how these combined approaches influence educational outcomes such as empathy, communication, professional identity development, and patient-centered care.

### Research questions

This review was guided by the following research questions:

1. How is narrative medicine integrated with digital tools in healthcare education contexts?
2. What are the reported educational outcomes of this integration in terms of empathy, communication, professionalism, and patient-centered care?

### Protocol and registration

This review was not pre-registered, and no protocol was published. We acknowledge this as a limitation and suggest future reviews adhere to protocol registration via PROSPERO or similar registries.

### Data sources and search strategy

We systematically searched several databases including PubMed, MEDLINE, and Google Scholar to ensure comprehensive coverage of the literature. Additional searches were considered in PsycINFO and Scopus to broaden scope, but these were ultimately excluded due to resource constraints; however, their absence is noted as a limitation. The search was tailored to include a combination of key terms aimed at capturing the broad spectrum of narrative medicine and its connection with technology in healthcare education.

The keywords used were: ‘narrative medicine’; ‘technology in healthcare education’; ‘patient-centered care’; and ‘medical training’. These terms were combined using Boolean operators to maximize the retrieval of relevant articles. Medical Subject Headings (MeSH terms] were not applied in the PubMed search. We acknowledge this as a limitation and encourage future reviews to incorporate controlled vocabulary to advance precision.

To refine the search, filters were applied to include only articles that were published in English and underwent a peer-review process. The rationale for excluding non-English articles was based on feasibility constraints and resource availability for translation. We recognize this as a potential source of selection bias. The search was conducted in January 2024, and no time frame limits were applied in the initial query to capture historical and current perspectives. Emphasis, however, was placed on more recent studies to reflect current practices and innovations.

### Eligibility criteria

The inclusion criteria encompassed studies that,

- Provided empirical evidence or were themselves systematic reviews.
- Focused on the integration and outcomes of narrative medicine within healthcare education.
- Evaluated the effects of narrative medicine on empathy, communication skills, professional development, and patient-centered care.

The exclusion criteria included,

- Articles not published in English.
- Studies not addressing educational outcomes.
- Commentaries, editorials, opinion pieces, or non-peer-reviewed articles.

### Study selection process

**Figure 1.**
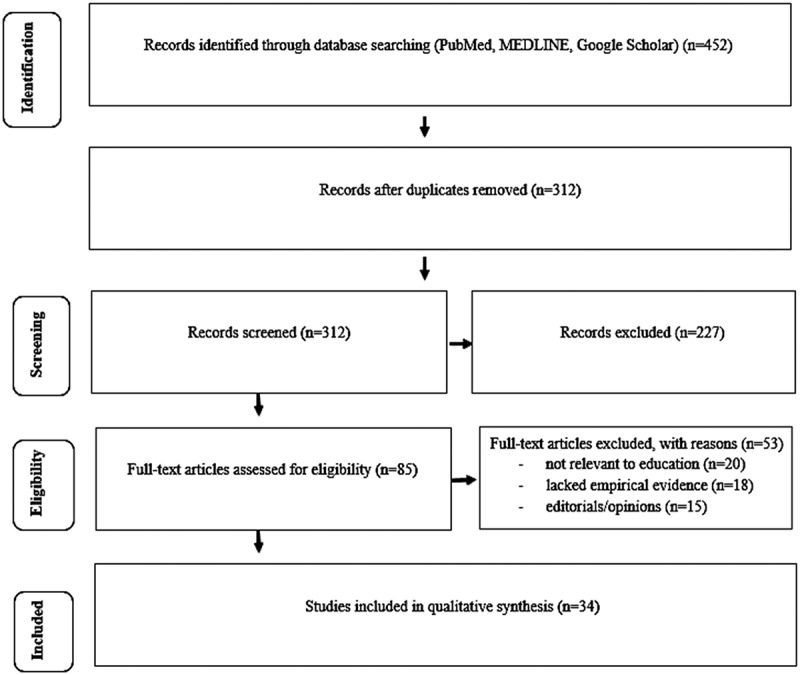
PRISMA 2020 flow diagram demonstrating the identification, screening, eligibility, and inclusion process for studies included in the systematic review.

Two reviewers independently screened titles and abstracts of the retrieved articles. Out of 452 initial records identified, 312 remained after duplicates were removed. A total of 85 full-text articles were assessed for eligibility, with 34 studies meeting all inclusion criteria. Disagreements were resolved by discussion or consultation with a third reviewer. The study selection process is demonstrated in the PRISMA 2020 flow diagram (Figure 1).

### Data extraction and quality assessment

For each of the 34 included articles, we extracted data on study context, research design, population, outcomes measured, and main findings. These characteristics are summarized in Table 1. Quality assessment was conducted using the Mixed Methods Appraisal Tool (MMAT) 2018, which is appropriate for appraising qualitative, quantitative, and mixed-methods studies. Two reviewers independently applied the MMAT to each study. Discrepancies were resolved through discussion or, when necessary, by consulting a third reviewer.

**Table 1.** Summary of included studies in the review (N = 34).

Author[s), year	Country/ setting	Study design	Population	Intervention/ tool	Outcomes measured	Key findings
[15]	Taiwan/Chung Shan Medical University	Quasi-experimental [mixed methods]	Health professions students	Narrative medicine intervention focusing on empathetic connection	Professional identity, reflective thinking, emotional catharsis, reflective writing competency	Improved professional identity, reflective thinking, emotional catharsis, and reflective writing skills.
[16]	China/Medical university [specific institution not specified]	Qualitative	Medical students	Narrative ability training	Empathy, communication skills	Enhanced empathy and communication skills, with improved doctor-patient relationships.
[17]	United Arab Emirates/Healthcare systems	Data analysis study	Healthcare systems	Big data integration	Personalized treatment, inventory efficiency	Big data improved treatment personalization and resource utilization, aligning with narrative care principles.
[18]	Iran/Isfahan University of Medical Sciences	Qualitative	135 Medical interns	Narrative medicine	Empathy, reflective capacity	Improved empathy and reflective capacity among students.
[19]	Singapore/Palliative care settings	Systematic scoping review	Healthcare professionals	Patient narrative analysis	Sociocultural insight, empathy	Narratives provided critical contextual insight, improving training in emotional and sociocultural care.
[20]	Medical education	Descriptive/Tool development	Medical students	Empathy and Clarity Rating Scale	Communication skill assessment	Developed a tool to quantify communication and empathy improvements via narrative training.
[21]	Clinical internships	Intervention study	Medical students	Narrative medicine during internship	Empathy	Significant empathy improvement compared to traditional teaching.
[22]	Nursing education	Randomized controlled trial [RCT]	Nursing students	Narrative medicine training	Empathy, academic performance	Improved OSCE scores, higher internship evaluations, and reduced empathy burnout.
[27]	Medical education	Review	Medical students	Narrative reflection	Critical thinking, empathy, decision-making	Reflection improves empathy, self-awareness, and clinical reasoning.
[35]	Iran/Shiraz and Fasa Universities of Medical Sciences	Qualitative content analysis	Undergraduate nursing students	Reflection in clinical practice	Emotional well-being, professional growth	Reflection improved emotional well-being and professional skills.
[24]	Australia/Two general hospital wards in New South Wales	Qualitative action research	Medical and surgical nurses	Modified Safewards interventions	Empathy, professional behavior, violence prevention	Narrative Safewards improved empathy and professional conduct in high-stress environments.
[28]	Health professions education	Descriptive	Healthcare professionals/students	Narrative medicine	Empathy, professionalism	Strengthened empathy and awareness of professional boundaries.
[26]	Oncology settings	Qualitative	Health professionals	Expressive writing	Stress, empathy	Expressive writing reduced stress and increased empathy in emotionally intensive care settings.
[25]	Undergraduate nursing education	Simulation-based intervention study	Nursing students	VR goggles with TeamSTEPPS	Communication, teamwork, empathy	VR enhanced clinical realism, improving communication and empathy by provideing multiple perspective simulations.
, [36]	Pregnancy care settings	Quantitative longitudinal study	Pregnant women and healthcare professionals	Mobile health communication app	Communication behavior, patient engagement	Mobile app improved professional-patient communication, patient engagement, and care personalization.
[23]	Hospital nursing handoffs	Descriptive linguistic analysis	Hospital nurses	Clinical decision support tools	Communication clarity, patient safety, professionalism	Digital tools improved handoff structure, reduced errors, and promoted empathy through improved understanding of patient needs.
[30]	IBD care	Mixed methods [digital and face-to-face interviews]	Patients and informal caregivers	Digital access to patient narratives	Communication, empathy, patient-centered care	Digital tools improved narrative accessibility, improving empathy and care personalization.
[31]	Multiple myeloma care	Intervention study [platform assessment]	Patients with multiple myeloma	All4Cure digital health platform	Patient activation, communication, narrative integration	All4Cure enabled rich medical histories and improved patient-provider communication.
[32]	USA/Clinical settings [unspecified]	Systematic review	Healthcare professionals and patients	Digital clinical workflow tools	Interaction time, empathy, diagnostic accuracy	Digital workflow optimization allowed more patient interaction and improved empathetic engagement.
[33]	Biomedical education	Curriculum innovation study	Biomedical students	Digital platforms for curriculum engagement	Reflective capacity, empathy, professionalism	Digital tools supported ethical reflection and narrative engagement, improving empathy and readiness for diverse care.
[34]	Higher education [non-healthcare specific]	Educational case study	University students and educators	Social media, online discussions, digital book talks	Reflective engagement	Digital forums promoted continuous reflection and experience sharing applicable to narrative medicine contexts.
[35]	Hospital nursing handoffs	Descriptive linguistic analysis	Nurses in hospital settings	Digital informatics platforms	Reflection, empathy, communication accuracy	Digital tools enhanced narrative precision and reflective practice in nursing handoffs, improving empathy and safety.
[37]	Educational technology for learning difficulties	Intervention study	Children with learning difficulties	Dyslexia-friendly eBooks	Content accessibility, learning outcomes	Digital tools adapted content for learner needs, suggesting similar benefits for narrative medicine education.
[38]	Social studies education	Digital game-based learning study	School students	Lazarus educational game	Engagement, learning outcomes	Game-based tools improved engagement and comprehension, indicating value for narrative medicine teaching.
[39]	Tertiary education	ICT integration study	University students	ICT platforms	Learning outcomes, digital literacy	ICT improved outcomes and skill acquisition, suggesting relevance for teaching narrative medicine.
[40]	Online healthcare service feedback	Content analysis	Patients using digital healthcare	Narrative patient feedback	Care quality, patient satisfaction	Narratives in digital feedback shaped evaluations and guided service improvements.
[29]	Electronic Health Records [EHR]	Narrative analysis	Healthcare professionals	EHR structure and use	Narrative flow, interprofessional communication	EHRs risk disrupting narratives but structured integration supports care continuity and collaboration.
[41]	Digital consultations	Case report	Patients and providers	Digital care platforms	Communication efficiency, patient safety	Digital platforms improved efficiency and access; narrative elements essential to preserve empathy.
[42]	Healthcare decision-making	Theoretical analysis	Clinicians and healthcare systems	Narratives & empirical data	Clinical decision-making quality	Narratives contextualized quantitative data, improving informed and empathetic decisions.
[43]	EHR development	System development	Clinicians	Narrative-capable EHR model	Data richness, care continuity	EHR model preserved clinician narratives, enabling better data reuse and care quality.
[44]	University of Michigan Medical School	Theoretical/educational program	Medical students	Narrative pedagogy [Family Centered Experience]	Empathy, patient-centeredness	Engagement with patient stories improved empathetic understanding and professionalism.
[45]	Higher education	Conceptual analysis	Students in technology-enhanced settings	Digital storytelling	Critical thinking, emotional engagement, digital literacy	Digital storytelling nurtured critical thinking and prepared students for tech-based healthcare.
[46, 47]	Interactive media and education	Theoretical framework	Learners in e-learning environments	Interactive digital narrative [IDN]	Narrative adaptability, learner engagement	IDN frameworks support immersive simulations and decision-based learning in clinical education.
[48]	Clinical education	Framework proposal	Health professions students	Caring Stories framework	Empathy, interprofessional collaboration	Patient narratives foster empathy and interdisciplinary learning in clinical education settings.

### Synthesis of results

A qualitative synthesis was conducted due to heterogeneity in methods and outcome measures. Emerging themes included:

- The effectiveness of narrative medicine in improving empathetic communication.
- The role of digital platforms in delivering narrative-based education.
- Professional identity formation through narrative reflection.

### Risk of bias

Risk of bias was considered through methodological quality ratings and narrative assessment. Strategies to reduce bias included independent screening by multiple reviewers and the use of standardized checklists.

## Results

### Theoretical insights and deductive reasoning

#### Narrative medicine’s role in contemporary healthcare education

The benefits of narrative medicine are well-documented across various studies. [15], found significant improvements in healthcare students’ reflective thinking, communication, and empathy through structured narrative medicine interventions. These findings are echoed by [16], who reported advanced narrative abilities in medical students, which bolstered their empathy and communication skills.

In tandem with these pedagogical strategies, the integration of digital tools such as electronic health records, mobile learning platforms, and data analytics has expanded the reach and efficiency of narrative-based education. The integration of big data into healthcare practices provides additional benefits, in the realm of personalized medicine. A recent study by [17], highlights how big data are used to optimize patient treatment and reduce inventory waste in healthcare settings. This study emphasizes the potential of big data to improve health outcomes, expanding the efficiency of healthcare delivery by tailoring treatments to individual patient needs, a core principle of narrative medicine. Further [18], stress the impact of narrative medicine on medical students in Iran, highlighting substantial advancements in reflective capacity and empathy. These traits are crucial for cultivating professionalism in medicine, suggesting that narrative medicine directly contributes to the development of core competencies needed in medical practice.

In specialized care contexts, such as palliative or oncology care, the integration of digital storytelling tools, such as patient video journals or voice-based platforms, has allowed for richer narrative engagement. [19], explored its role in palliative care, demonstrating how patient stories provide critical psycho-emotional, sociocultural, and contextual insights that are overlooked in conventional medical training. This study supports the necessity for medical curricula to incorporate effective training in interpreting and applying patient narratives in clinical settings. The introduction of the Empathy and Clarity Rating Scale by [20], provides an innovative metric to assess the impact of narrative and improvisational training on communication skills in medical education. This tool highlights the quantifiable benefits of integrating narrative practices into training programs. [21], investigated the integration of narrative medicine into clinical internships, observing advancements in medical students’ empathy compared to traditional teaching methods. This demonstrates the utility of combining narrative education with experiential, digitally supported clinical placements, such as virtual simulation or e-portfolio reflection tools, further amplifying empathy and reflective engagement.

A randomized controlled trial by [22], revealed that narrative medicine improves empathic abilities and academic performance among nursing students, emphasizing the broad applicability of narrative techniques beyond medical training to include other healthcare disciplines. One of the standout findings from the study was the marked improvement in students’ empathy levels, among those who underwent both theoretical and practical narrative medicine training. This advancement in empathy was facilitated through engaging activities such as exchanging diaries with patients and reflective writing, allowing students to connect with and understand patient experiences. Such training helps students develop an appreciation of patients’ emotional and psychological states, which is critical for effective patient-centered care.

The integration of narrative medicine into the curriculum also had a positive impact on students’ academic achievements and clinical skills, as evidenced by improved performance in objective structured clinical exams [OSCEs] and higher internship evaluations. OSCE performance was measured using a structured rubric assessing clinical communication, empathy, and diagnostic accuracy, while internship evaluations were based on supervisors’ assessments of students’ professionalism, teamwork, and responsiveness to patient needs. These results suggest a multidimensional benefit; narrative medicine nurtures emotional intelligence and advances procedural competencies. The study also highlighted the importance of continuous narrative medicine education to prevent empathy burnout, a common challenge in nursing careers. Integrating narrative strategies in digital professional development modules provides a sustainable approach to long-term empathy maintenance.

Supporting the development of reflective practitioners [23], emphasized the importance of narrative methods in nurturing critical assessment skills in students, essential for continuous professional development. The authors explore the critical role of reflection in medical education by reviewing various concepts, models, principles, and teaching methods. Their study emphasizes the importance of integrating reflection into medical curricula to advance students’ critical thinking, clinical reasoning, and decision-making skills. Reflective practices are shown to improve self-awareness, empathy, and a comprehensive understanding of professional responsibilities. The authors suggest that reflection enables medical students to better manage the complexities and emotional demands of clinical practice, nurturing personal growth and professional development. They advocate for medical schools to create supportive environments that encourage routine reflective practice, producing more competent and empathetic physicians. This study highlights reflection as an essential element for advancing medical education and enriching the training of future healthcare providers.

Similarly [24], found that narrative practices deepen students’ understanding of clinical experiences, leading to advanced patient care. The study explores the perceptions of Iranian nursing students on the impact of reflection during their clinical practices. Utilizing in-depth interviews with 20 students, the research identified personal and professional benefits from reflective practices. Professionally, students noted improvements in their nursing functions, advanced ability to share and generalize experiences across different scenarios, encouraging a movement toward greater professionalism. On a personal level, reflection led to increased inner satisfaction and peace of mind, contributing to better emotional well-being. These findings highlight the dual value of reflective practices, improving clinical skills and adaptability and promoting emotional health, which is vital for effective patient care. The study advocates for nursing education to incorporate and support reflective practices, thereby nurturing more competent and emotionally resilient healthcare professionals.

Through the employment of patient stories into training, advanced through digital narrative capture and virtual storytelling environments, narrative medicine improves the communication skills and empathy of healthcare providers and advances their clinical practice. This ensures that future healthcare professionals are equipped to deliver compassionate, patient-centered, and reflective care, bridging the gap between medical science and the complex narratives of patient lives. By integrating digital innovations with narrative practices, healthcare education nurtures a generation of providers who are clinically proficient and attuned to the lived experiences of those they serve.

### Integration of narrative medicine and digital tools in developing empathy and professionalism in healthcare

Recent research emphasizes the impact of integrating narrative medicine and digital tools on improving empathy and professionalism among healthcare providers. A series of studies demonstrate how this integration facilitates a deeper understanding of patient experiences and improves communication skills, which are crucial for patient-centered care.

[25] explored medical and surgical nurses’ experiences with implementing contextually adapted Safewards interventions into hospital wards. The study highlighted how narrative-driven, contextually embedded interventions advanced through structured storytelling and digital tracking mechanisms improved nurses’ professional practices and empathy toward patients. By incorporating patient and staff narratives into Safewards, the study found an advancement in nurses’ ability to manage violence and engage empathetically with patients, demonstrating the powerful role that narrative-informed and digitally structured environments play in shaping professional behavior and emotional regulation in high-stress clinical settings.

Building on the theme of narrative integration [26], investigated the influence of narrative medicine within health professions education. Their research revealed that the structured use of narrative practices, supported by journaling platforms and digital feedback systems, strengthens empathetic connections healthcare professionals make with their patients and recognize their professional limits. This dual benefit is central for nurturing personal growth and professional excellence in healthcare settings, suggesting that technology-advanced narrative approaches enrich health professions education by deepening empathy and strengthening professional identity.

Further emphasizing the therapeutic benefits of narrative practices, a study by [27], examined the impact of expressive writing for health professionals working with oncology patients. They found that narrative medicine, through expressive writing, serves as a valuable tool for managing stress and increasing empathy among healthcare professionals. The use of online platforms to facilitate reflective writing and narrative sharing created a safe and accessible space for self-expression, reinforcing emotional resilience in high-pressure settings. This approach supports professional training and improves the quality of care provided to patients, in emotionally charged fields like oncology. The study advocates for the inclusion of narrative and expressive writing in professional training to nurture a healthcare environment that values emotional well-being and professional development.

### Digital tools streamlining communication and training processes

Recent advancements in digital technologies are being leveraged to advance communication and training processes in healthcare, contributing to the professional development and empathetic interactions of healthcare providers. The integration of innovative tools and methodologies streamlines educational and operational processes, enriching the quality of care provided to patients.

[28] investigates the use of virtual reality (VR] goggles combined with the TeamSTEPPS methodology in undergraduate nursing education. This research accentuates the potential of VR technology to revolutionize healthcare education. By immersing nursing students in realistic clinical environments, VR enables them to practice and refine their communication and teamwork skills in a controlled setting. This method bridges the gap between theoretical knowledge and practical skills, preparing students for real-world challenges in patient care and team collaboration. Moreover, VR simulations allow learners to adopt the perspectives of various stakeholders, e.g., patients, physicians, and nurses, thereby nurturing a multidimensional understanding of care and improving empathy. The study stresses that such immersive digital training tools are effective in shaping professional behavior and empathetic responsiveness.

Building on the theme of digital facilitation in communication[36], evaluate the effectiveness of a mobile app designed for pregnant women. This quantitative longitudinal study highlights how digital tools, designed apps, improve communication behaviors between healthcare professionals and patients. The app serves as a continuous link between the two, providing a platform for transmitting vital health information, scheduling reminders, and providing educational content. The study shows that this mobile app advances real-time communication and information retention and promotes patient engagement, satisfaction, and emotional connection, as core aspects of empathetic, personalized care. It demonstrates how mobile health technology empowers healthcare professionals to maintain patient-centered relationships while improving operational efficiency.

Furthermore, [29], explore the linguistic aspects of nursing handoffs in varied-acuity hospital settings. The research emphasizes the critical role of digital tools in improving the clarity and effectiveness of these handoffs, which are essential for patient safety. The implementation of clinical decision support systems and technology to streamline communication, healthcare units reduce the potential for errors. The study indicates that structured, tech-supported handoffs nurture a culture of professionalism, allowing nurses to communicate more thoroughly and empathetically by better understanding and anticipating patients’ individual needs. These findings highlight how even back-end digital systems indirectly but meaningfully support humanistic, emotionally intelligent care delivery. Thematic patterns emerging from the synthesis of these studies are summarized in Table 2.

**Table 2.** Thematic synthesis of outcomes by intervention.

Outcome theme	Tools/interventions used	Example studies
Communication skills	EHR structure and use, All4Cure digital health platform, Mobile health communication app, Digital care platforms, Empathy and Clarity Rating Scale, Clinical decision support tools	[Kötting et al., 2024, 20, 31, 35, 35, 41]
Empathy	Digital informatics platforms, Narrative reflection, Narrative medicine during internship, Narrative medicine training, Expressive writing, Narrative-driven Safewards, Patient narrative analysis, Narrative medicine, Narrative medicine interventions, Digital clinical workflow tools, Narrative pedagogy [Family Centered Experience], Caring Stories framework, Narrative ability training, Digital access to patient narratives, Digital platforms for curriculum engagement, VR goggles with TeamSTEPPS	[15, 16, 18, 19, 21–23, 25–28, 30, 32, 33, 35, 44, 48]
Professionalism	Reflective practice	[24]
Reflective practice	Social media, online discussions, digital book talks	[34]
Technology-enhanced learning & patient-centered outcomes	ICT platforms, Dyslexia-friendly eBooks, Lazarus educational game, Digital storytelling, Big data integration, Interactive digital narrative [IDN], Narrative-capable EHR model, Narrative patient feedback, Narratives & empirical data	[37–39, 46, 47, Niemi & Multisilta, 2016, 17, 40, 42, 43]

### Synthesis of theoretical benefits

#### Greater accessibility to patient histories and narratives in real-time

Recent studies highlight the redefined role that digital tools play in improving the accessibility and effectiveness of narrative medicine in healthcare settings. By integrating technology into the management of patient histories and narratives, these tools provide great benefits in diagnostic accuracy and empathetic patient care.

[30], investigate the value that patients and informal caregivers place on accessible communication within the context of inflammatory bowel disease [IBD] care. Their research, which involved face-to-face and digital interviews, highlighted how digital tools facilitate the easy and efficient access to patient narratives. The study demonstrated that digital platforms enable providers to retrieve detailed accounts of patient experiences in real-time, which contributes to a more nuanced understanding of patient needs. The findings suggest that improving narrative accessibility through digital means improves understanding and empathy among healthcare providers, leading to more patient-centered care.

Similarly [31], explored the impact of the All4Cure health technology intervention on patient activation in multiple myeloma. Published in Blood, their study assesses a platform that integrates comprehensive patient medical records to create a detailed narrative of the patient’s medical history. This platform facilitates better communication between patients and their care teams, improving care outcomes by making medical histories more accessible and interpretable. The integration of structured, narrative-based data within digital platforms supports collaborative decision-making and deepens provider engagement with patient stories. Such digital interventions exemplify how technology advances the narrative aspect of medicine, improving diagnostic processes and empathetic interactions.

Furthermore [32], discuss the use of digital tools to give patients focused attention. Their study explores how technology streamlines clinical workflows, such as the recording of patient histories and examinations. By reducing time spent on administrative tasks and improving the flow of information, digital tools allow healthcare professionals to redirect their focus toward more meaningful patient interactions. This facilitates deeper empathetic connections and more accurate diagnoses. Increased provider availability for active listening and personalized care is shown to directly contribute to improved clinical outcomes and greater patient satisfaction. Accessible patient histories and improved communication between patients and providers enable healthcare professionals to integrate empathy and professionalism more fully into their practice. This supports a broader shift toward a model of care that is data-driven and human-centered.

### Opportunities for reflective practice through digital logs or forums

In healthcare education, the integration of digital tools with narrative medicine provides promising avenues for improving reflective practices among healthcare professionals. Recent studies have begun to explore how these technologies support and enrich the narrative components of medical training, developing greater empathy and professionalism.

[33], emphasize the potential of digital platforms to support reflective practices in a biomedical educational context. The authors explore how creative tools are utilized to design an inclusive curriculum that bridges the digital divide, enabling students to engage more deeply with the narrative aspects of patient care. Their findings suggest that structured reflection through digital media, such as asynchronous discussion boards or multimedia storytelling platforms, facilitate ethical reasoning and greater awareness of humanistic care. Through online platforms, students are provided with a space to reflect on complex medical ethics and patient stories, which is critical for developing a thorough understanding of the human elements in healthcare. This approach advances students’ empathy and professionalism and equips them to handle diverse patient needs effectively.

Building on the idea of using digital platforms for reflective education [34], provide further insights into how digital environments are adopted in educational settings beyond healthcare. They discuss how social media, online discussions, and digital book talks improve reflective practices in higher education. While the focus is not exclusively on healthcare, the principles outlined are readily applied to narrative medicine. These platforms nurture dialogic reflection, community-building, and long-term narrative competence through peer exchange. Ongoing discussions and reflections among healthcare professionals through digital forums promote a continuous exchange of experiences and insights. Such interactions improve understanding and empathy among healthcare providers, as they share and reflect on their patient interactions, thus strengthening their narrative competence and professional development.

Similarly [35], examine the use of digital tools and informatics to refine the narrative and linguistic aspects of nursing handoffs in varied-acuity hospital settings. The study highlights how digital platforms advance the precision and effectiveness of these communications, promoting a reflective practice among healthcare professionals. Structured digital templates and documentation tools encouraged narrative coherence and reflection-in-action during clinical communication. Through more structured and detailed patient information transfer, these tools improve patient safety, developing a deeper understanding and empathy among nurses. This reflective practice, facilitated by digital advancements, highlights the role of technology in increasing the narrative competence of healthcare workers and contributing to better patient outcomes.

### Indirect evidence and inferences

#### Extrapolating benefits of digital tools for narrative medicine education

Recent studies have demonstrated the efficacy of digital educational tools in improving learning outcomes across various educational settings, suggesting potential benefits for their application in narrative medicine education as well.

For instance [37], explored the impact of eBooks with dyslexia-friendly features on children with learning difficulties. The research highlighted how these digital tools facilitate learning by adapting content to meet specific learner needs. These findings highlight the potential for digital platforms in narrative medicine education to advance accessibility and student engagement, when addressing complex or sensitive clinical narratives. Through tailored content presentation, such technologies enable learners to grasp the subtleties of patient experiences and ethical dilemmas effectively.

In exploring digital advancement in education [38], investigated the effects of the Lazarus educational game on learning outcomes in social studies. The study revealed that incorporating digital games and other technology-driven tools improved student engagement and comprehension. This suggests that serious games or gamified simulations are applied in narrative medicine curricula to cultivate empathy, communication, and ethical reasoning in an immersive and student-centered format.

Moreover [39], studied the impact of Information and Communication Technology [ICT] integration in tertiary education, on improving language learning outcomes. The findings indicate that effective ICT integration improves learning outcomes and equips learners with essential skills for success in a digital era. This reinforces the potential of ICT tools, such as digital storytelling apps, reflective blogging platforms, or e-portfolio systems, to strengthen narrative medicine instruction by improving communication, critical reflection, and digital fluency. Making educational content more accessible and engaging benefit narrative medicine pedagogy, where learners grasp complex concepts and apply them effectively in their future professional practice.

#### Introducing narrative elements into digital platforms and qualitative improvements in patient care or provider satisfaction

The integration of narrative elements into digital healthcare platforms is recognized for its potential to advance patient care and provider satisfaction. This transition toward digitalization, while boosting efficiency and precision in healthcare, risks the loss of personal patient-provider interactions, for empathetic and effective care.

[40], analyzed patient feedback on healthcare services provided online. The researchers found that narratives are influential in shaping care evaluations, especially in instances of negative feedback. Their findings demonstrate that digital systems that incorporate narrative feedback mechanisms advance the responsiveness of healthcare services and support real-time quality improvements. Further exploring the impact of digital tools on healthcare narratives [29], examined the challenges posed by electronic health records [EHRs] to the narrative construction of patient care. Their findings indicate that while EHRs disrupt the flow of narrative information, maintaining a structured narrative within digital records is central for comprehensive patient care and effective interprofessional communication. The study supports the development of narrative structured EHR templates that preserve clinical storytelling while enabling efficient documentation.

Similarly, [41], reported on the introduction of digital platforms for patient-provider consultations, highlighting improvements in communication efficiency and flexibility. This study highlights the benefits of digital tools in improving access to care and patient safety, while also pointing to the necessity of integrating narrative elements to preserve the human aspect of healthcare interactions. The use of video-based consultations and patient journaling platforms complement these systems, strengthening empathetic connection and reflective care practices.

Addressing the broader implications of narrative integration, [42], argued for the importance of combining narrative methods with empirical data in healthcare decision-making. Their research suggests that narratives contextualize the quantitative data provided by digital health technologies, thus supporting more informed and empathetic clinical decisions. Such hybrid approaches provide a framework for integrative care, where evidence-based practice is enriched through patients’ lived experiences. Lastly, [43], developed an electronic health record model that facilitates the rapid capture of detailed narrative observations. This model aims to preserve the richness of clinician narratives within a digital format, augmenting the reuse of medical data and improving the continuity and quality of patient care. Their findings point to the viability of narrative-aware informatics models that align data integrity with humanistic practice.

#### Theoretical frameworks and models that support the integration of narrative and digital approaches in medical education

In medical education, the integration of narrative and digital approaches presents a promising method for improving the technological proficiency and empathetic capacities of healthcare professionals. Several theoretical frameworks and models provide a robust foundation for understanding how these integrations are implemented to expand empathy and professionalism among medical students.

A foundational concept in this integration is narrative pedagogy, which emphasizes the use of illness narratives to cultivate empathy and professionalism. [44], articulated how such narratives deepen students’ understanding of patients’ experiences, thereby improving patient-centered care. One well-established example is the Family Centered Experience at the University of Michigan Medical School, where students engage directly with patients’ stories through home visits, reinforcing emotional insight and person-centered engagement. This model exemplifies how experiential storytelling supports the development of clinical empathy.

Complementing narrative pedagogy, digital storytelling provides a dynamic method for engaging students in their education. Niemi and Multisilta (2016) discuss the benefits of digital storytelling in nurturing critical thinking and emotional engagement through the creation of interactive narratives. They argue that digital narrative construction supports reflection and facilitates technological literacy, two skills critical in modern healthcare. This method supports the acquisition of core competencies while preparing students to function well in technology-rich environments.

Expanding on digital storytelling, the work of [46,47], introduces a framework for interactive digital narratives [IDNs] that emphasizes adaptability and user interaction. In medical education, these principles support the creation of branching simulation scenarios in which students must make real-time decisions, replicating the uncertainty and ethical complexity of clinical encounters. Such digital platforms allow learners to navigate through varied patient interactions, shaping outcomes based on their inputs, and promoting immersive, decision-based learning.

Complementary to the integration of narratives into medical training, [48], propose the Caring Stories framework, which utilizes patient stories to advance clinical education across healthcare disciplines. This model supports empathy development, facilitating interprofessional dialogue, and improving collaboration between future physicians, nurses, and allied health professionals. It provides a flexible structure for embedding narrative-based reflection across curricula.

The results derived from these frameworks suggest promising synergies between narrative medicine and digital technology in developing empathy and professionalism in medical education. However, it is important to recognize the speculative nature of these conclusions, given the current scarcity of large-scale empirical studies testing these integrations in clinical education contexts. While the theoretical underpinnings are conceptually strong, empirical validation remains a key next step. Future studies test the effectiveness of these integrative approaches through rigorous outcome-based evaluations. This would substantiate the theoretical claims and inform the design of medical education curricula that thoughtfully combine narrative and digital methodologies.

### Synthesis of results

The synthesis of results from integrating digital tools with narrative methods in medical education reveals a synergistic and mutually reinforcing relationship that enriches the training of healthcare professionals. This integrated approach is fundamental in developing more empathetic, professional, and technologically adept healthcare providers.

Narrative medicine promotes empathy by immersing students in patient stories and personal experiences [22, 44], encouraging them to adopt the perspectives of those they serve and understand the contextual dimensions of illness. Digital tools expand this narrative exposure by providing access to multimedia patient testimonies, digital storytelling repositories, and immersive virtual reality (VR) scenarios [27,28]. Such technologies enable learners to explore a broad spectrum of human conditions and sociocultural contexts, thereby broadening their empathetic and cross-cultural competencies.

Moreover, effective communication is foundational in healthcare, and narrative methods are instrumental in training students to listen attentively and respond empathetically [15,23]. Digital platforms advance this training by simulating real-time consultations, peer feedback environments, and AI-assisted communication analysis [28,35]. Interactive simulations and role-playing scenarios allow students to hone their communication skills, while integrated analytics provide detailed, actionable feedback that refine verbal and non-verbal aspects of interaction.

Professionalism in healthcare, characterized by accountability, ethical sensitivity, and a commitment to reflective practice, is also cultivated through narrative methods [1,24]. These approaches encourage students to confront and reflect on ethical dilemmas and professional behaviors through real-life narratives. Digital environments further support this development by provideing tools such as e-portfolios, discussion forums, and structured reflection modules [33,49]. These platforms facilitate longitudinal reflection and professional identity formation, while enabling peer mentorship and faculty guidance.

As healthcare relies on technology, from electronic health records (EHRs) to telemedicine, it is vital that providers are empathetic and technologically proficient. Integrating digital tools into medical training builds this proficiency, demonstrating how narrative data are harmonized with clinical records to enrich care [29,41,43]. This includes the use of narrative-advanced EHRs and teleconsultation systems that maintain human-centered interactions while maximizing efficiency. The adaptability of narrative-digital integrations across face-to-face, blended, and fully online environments strengthens their utility in global education contexts [34,39]. This flexibility improves accessibility, accommodates diverse learner needs, and supports inclusive education. As demonstrated by the reviewed studies, the integration of narrative and digital strategies is supported by emerging empirical and conceptual work across diverse healthcare and educational contexts. Implementing this holistic approach in healthcare education globally elevates the quality of training and patient care. The combined narrative-digital framework provides a scalable and evidence-informed strategy for curriculum development, instructional design, and faculty development, positioning medical education to meet the complex demands of 21st-century healthcare.

### Challenges and future directions

Despite its potential, the integration of narrative and digital approaches in medical education faces several challenges and suggests distinct opportunities for future research. A major obstacle is resistance from traditional educators who may remain skeptical of the capacity of digital tools to convey the humanistic dimensions of narrative medicine. This resistance stems from pedagogical conservatism, unfamiliarity with digital modalities, and a perceived divide between emotional learning and technological tools.

One challenge is institutional reluctance to adopt novel teaching modalities. [50], features the tension between static theoretical instruction and the dynamic, practice-oriented skills demanded by contemporary clinical environments. The author advocates for immersive digital narratives to bridge this theory-practice gap, suggesting that interactive educational technologies provide context-rich and clinically relevant learning experiences.

Technical barriers also pose constraints. Implementing digital infrastructure that supports narrative-based training requires substantial investment in hardware, software, and human resources. [49], describes a pilot program in which medical students used digital storytelling to reflect on clinical experiences, noting that while student outcomes were positive, sustained support and training were essential for success. In addition, [51], highlight integration issues in patient education systems, emphasizing the complexities of narrative components into existing digital health workflows. [1], draw attention to the limited empirical evidence on the long-term impact of narrative medicine on learner behavior and patient outcomes. Without robust data, institutional stakeholders may be hesitant to fully commit to these methods. Yet, there are promising directions. [52], demonstrate that digital storytelling advances reflective learning and student engagement. By aligning with the digital fluency and reflective inclinations of modern learners, these tools may overcome resistance and support sustained behavioral change in clinical practice.

Future research needs to focus on empirically evaluating the educational outcomes of integrated narrative-digital models, their effects on empathy development, communication competence, ethical decision-making, and long-term professional behavior. Randomized trials, mixed-method evaluations, and longitudinal studies provide critical visions into the efficacy and durability of these interventions. Despite these challenges, the integration of narrative and digital approaches enable deeper engagement, emotional intelligence, and critical reflection, preparing healthcare professionals to deliver care that is technologically sophisticated and humane and shape the next generation of empathetic and tech-savvy clinicians.

## Discussion

This review has synthesized key understandings from various studies, featuring the impact of integrating digital and narrative medicine into healthcare education. The evidence demonstrates that this integration enriches students’ cognitive and emotional engagement and augments clinical competencies, ethical awareness, and digital fluency. This integration extends the capabilities of future healthcare providers to engage in more empathetic and effective patient care.

The benefits of narrative medicine, as highlighted by studies of [15,16], demonstrate substantial improvements in healthcare students’ empathy, communication, and reflective thinking. These outcomes support the central premise that narrative training cultivates humanistic awareness, which is critical for person-centered care. These advancements nurture a deeper understanding of patients’ experiences and cultivating essential professional skills. Similarly, the integration of big data, as discussed by [17], extends the efficiency of healthcare delivery, allowing for more personalized treatment approaches that align with the core principles of narrative medicine. Their findings highlight that data-driven personalization coexist with narrative empathy, providing a model for compassionate precision medicine.

Further reinforcing the value of narrative medicine, research by [18,19], demonstrates how narrative practices influence medical training and patient care, in specialized contexts like palliative care. These studies show that narrative medicine improves clinical skills and advances the relational aspects of caregiving, providing healthcare professionals with the tools to better understand and address the psycho-emotional and sociocultural dimensions of patient care. This dual function, technical and relational, is vigorous in complex, high-stakes care environments.

The introduction of the Empathy and Clarity Rating Scale by [20], and the empirical research on narrative medicine’s practical applications in clinical internships by [21], further demonstrate the tangible benefits of embedding narrative practices in medical training. These studies confirm that empathy is taught and measured, validating narrative-based education as a replicable and scalable intervention. These innovations highlight the practical advantages of narrative medicine, suggesting that such educational strategies advance the empathic abilities and academic performance of healthcare students.

This review also touched upon the promising role of digital tools in supporting narrative medicine. The studies presented indicate that digital platforms advance the accessibility of patient histories, provide greater opportunities for reflective practice, and improve communication and training processes within healthcare settings. Tools such as VR simulations, mobile health apps, e-portfolios, and digital storytelling environments, enable educators extend narrative learning across diverse settings and learning styles. These technological advancements streamline the integration of narrative elements into healthcare, potentially leading to qualitative improvements in patient care and provider satisfaction.

Taken together, the integration of digital and narrative medicine in healthcare education represents a pedagogically innovative and future-facing approach. It promises to improve professional development and patient outcomes by supporting a generation of healthcare providers who are adept at clinical and narrative competencies. This integration responds to the evolving demands of modern medicine for future providers to deliver care that is technologically proficient and humane.

Nevertheless, this review is subject to certain limitations. While it draws on a broad and conceptually rich set of studies, much of the existing research is qualitative or limited in scale. The lack of longitudinal or controlled empirical evaluations makes it difficult to determine the long-term impact of these educational strategies on actual clinical behavior and patient outcomes. Moreover, most available studies originate from specific academic or geographic contexts, which may limit generalizability. As outlined in Table 3, key barriers, enablers, and research needs were synthesized to guide the implementation of narrative and digital integration in healthcare education.

**Table 3.** Summary of barriers, enablers, and future directions for integrating narrative and digital approaches in medical education.

Category	Theme	Descriptive notes
Barriers	Resistance from educators and institutions	Traditional mindsets may be skeptical of digital/narrative methods
	Lack of empirical outcome-based evidence	Limited data showing long-term impact on student outcomes
	Technical infrastructure and training challenges	Need for investment in digital tools and training support
	Difficulty integrating into traditional curricula	Misalignment between narrative approaches and existing curriculum structures
Enablers	Positive learner engagement with digital tools	High student satisfaction with VR and reflective tools
	Flexibility of narrative methods across platforms	Narrative medicine adaptable to in-person and digital contexts
	Pedagogical innovation in medical schools	Some programs already experimenting with hybrid approaches
	Supportive policy or institutional interest in humanistic education	Growing emphasis on empathy and professionalism in accreditation frameworks
Research needs	Longitudinal studies on clinical impact	Measure how narrative-digital models affect real-world practice and empathy retention
	Comparative studies vs. traditional methods	Assess which model best supports skill development and learner satisfaction
	Integration strategies in interprofessional education	Examine how narrative practices work across healthcare disciplines
	Research on faculty development and training effectiveness	Explore how to upskill faculty to deliver integrated content effectively

Future research may empirically examine the effectiveness of integrated narrative-digital curricula across medical schools and health training institutions. This includes randomized trials, comparative studies with traditional teaching models, and mixed-method evaluations focusing on empathy retention, communication skills, and professional identity development. Researchers might also explore faculty readiness, cost-effectiveness, and the impact on interprofessional collaboration. Finally, integration efforts extend beyond curriculum design and include institutional policies, accreditation standards, and faculty development programs. Integrating narrative and digital competencies into national medical education frameworks is necessary for sustaining innovation and ensuring that healthcare education remains responsive to technological change and the humanistic core of clinical care.

## Conclusion

This review examined narrative and digital medicine within healthcare education, providing a synthesis of theoretical frameworks, empirical findings, and applied innovations. The evidence highlights the potential of integrating these approaches to develop empathy, communication, clinical reasoning, digital literacy, and professional identity formation among healthcare learners. Digital platforms augment the delivery and accessibility of narrative practices through simulations, storytelling tools, reflective media, and electronic health record (EHR) enhancements, creating a conceptual roadmap for a medical education that is technologically advanced and humanistically grounded. Narrative medicine, when supported by digital tools, cultivates healthcare professionals who are clinically competent and attuned to the lived experiences of their patients.

Despite these promising developments, further empirical research is needed to validate the long-term educational and clinical impacts of these integrative approaches. Future studies might examine behavioral, ethical, and patient-centered outcomes across diverse institutions and healthcare settings. Rigorous evaluation will be cardinal to redefine these conceptual visions into evidence-based curricular models for widespread adoption.

The findings of this review carry important implications for medical educators, curriculum designers, and policymakers. Institutions are encouraged to entrench narrative-digital competencies into core learning outcomes, faculty development programs, and accreditation standards. This dual emphasis on empathy and technological agility represents a strategic direction for preparing future-ready clinicians in a complex and evolving healthcare system.
